# Complete Genome Sequences of Four Virulent Rabies Virus Strains Isolated from Rabid Animals in Russia

**DOI:** 10.1128/genomeA.00140-13

**Published:** 2013-05-09

**Authors:** E. M. Poleshchuk, A. A. Deviatkin, V. G. Dedkov, G. N. Sidorov, J. V. Ochkasova, I. A. Hodjakova, I. A. Schukina, S. I. Savel’ev, A. G. Golenskih, G. A. Shipulin

**Affiliations:** Institute for Natural Foci Infections, Omsk, Russiaa;; Central Research Institute for Epidemiology, Moscow, Russiab;; Omsk State Pedagogical University, Omsk, Russiac;; Management of Federal Service on Customers’ Rights Protection and Human Well-Being Surveillance in Lipetsk Region, Lipetsk, Russiad;; North-West State Medical University, St. Petersburg, Russiae;; Management of Veterinary Science in Lipetsk Region, Lipetsk, Russiaf

## Abstract

Rabies virus (RABV) strains Rus(Lipetsk)-8052f, Rus(Lipetsk)-8053c, Rus(Lipetsk)-8054f, and Rus(Lipetsk)-8057f were isolated from foxes (*Vulpes vulpes*) and a cat (*Felis catus*) in the Lipetsk region of Russia in 2011. Close relationships between these strains and the members of the “Cosmopolitan” group from Russia (98% homology) and from Europe (95% homology) were estimated.

## GENOME ANNOUNCEMENT

Rabies virus (RABV) has circulated in the territory of Russia since the beginning of time. The incidence of rabies was observed in populations of wolves and dogs many centuries ago. Outbreaks of rabies occur periodically in the fox population. The incidence of rabies among the fox population was absent in the Russian Empire and the Soviet Union from the end of the 19th century until World War II. However, since the 1940s, epizootic outbreaks of rabies among foxes have been observed both in the former Soviet Union and in Western Europe. Currently, this fox-associated epizootic appears in northern Eurasia (1–3). In Russia, active complex investigations into the ecological, epidemiological, epizootic, and virologic characteristics of the natural foci of rabies associated with red foxes were carried out during the last half of the 20th century (4–7). For a long time, the population of RABV was thought to be homogenous. However, estimating the genetic diversity for RABV became possible after methods of molecular typing were developed and the genome was sequenced (8–11).

In 2011, for the analysis of the molecular diversity of RABV, four strains were isolated in the Lipetsk region in Russia (three strains from rabid foxes and one strain from a rabid cat). All strains were isolated from rabid animals by intracerebral mouse inoculation (12). The samples were initially identified by the direct fluorescent-antibody test (13) and the rapid rabies enzyme-linked immunosorbent assay (14).

The strains were named Rus(Lipetsk)-8052f, Rus(Lipetsk)-8053c, Rus(Lipetsk)-8054f, and Rus(Lipetsk)-8057f.

The complete genome was generated by reverse transcription-PCR using 18 pairs of primers for amplifying 18 overlapped fragments of RABV. The PCR products were purified and sequenced using an ABI Prism 3500 XL (Applied Biosystems) DNA analyzer. Complete genomes were assembled using the CLC Genomics Workbench (version 5.5.1). The protein sequences were assembled using MEGA (version 5) (15).

The complete genomes of the strains are 11,797 nucleotides (nt). The lengths of the coding areas are 1,353 nt for the N gene, 891 nt for the P gene, 609 nt for the M gene, 1,775 nt for the G gene, and 6,384 nt for the L gene. Comparison between the complete genomes allowed us to determine the nucleotide identity at a level of 98 to 99%. Close relationships with the members of the “Cosmopolitan” group (16, 17) from Russia (unpublished data) and from Europe (18) were also discovered (nucleotide identities of 98% and 95%, respectively). The levels of identity to other cosmopolitans that are isolated in Africa, America, and Asia are 85 to 95%.

Complete genome analysis might play an important role in further investigations into the epidemiology and pathogenesis of RABV.

### Nucleotide sequence accession numbers.

The complete genome sequences of RABV strains Rus(Lipetsk)-8052f, Rus(Lipetsk)-8053c, Rus(Lipetsk)-8054f, and Rus(Lipetsk)-8057f have been deposited in GenBank under the accession no. KC595280, KC595281, KC595282, and KC595283, respectively.
